# Application of artificial intelligence in palliative care: a bibliometric analysis of research hotspots and trends

**DOI:** 10.3389/fmed.2025.1597195

**Published:** 2025-05-21

**Authors:** Mingxia Pan, Renling Huang, Chenxi Liu, Yuanfang Xiong, Na Li, Huan Peng, Yongqi Liang, Weisheng Gu, Hanjiao Liu

**Affiliations:** ^1^School of Nursing, Fujian University of Traditional Chinese Medicine, Fuzhou, China; ^2^Shenzhen Hospital of Integrated Traditional Chinese and Western Medicine, Shenzhen, China

**Keywords:** artificial intelligence, palliative care, nursing care, bibliometric analysis, visual analytics

## Abstract

**Background:**

Palliative care, essential for improving quality of life in patients with serious illnesses, faces challenges such as resource limitations, workforce shortages, and the complexity of personalized care. AI’s capabilities in data analysis and decision-making offer opportunities to optimize symptom management, predict end-of-life risks, and tailor care plans. However, existing research emphasizes isolated AI technologies rather than systematic evaluations of its developmental trajectory in palliative care, particularly through bibliometric and visualization studies. This gap obscures trends in technological applications, interdisciplinary collaboration pathways, and research hotspots, potentially hindering AI’s practical innovation in the field.

**Objective:**

This study employs bibliometric methods to analyze research trends in AI-driven palliative care, mapping knowledge structures and identifying hotspots to inform future advancements.

**Methods:**

Data from the Web of Science Core Collection (inception to February 28, 2024) were analyzed using HistCite for bibliometric aggregation, VOSviewer for co-occurrence analysis, and CiteSpace for keyword trends.

**Results:**

Among 246 publications from 45 countries, 615 institutions, and 1,456 authors, output surged notably between 2020 and 2024. The U.S. and the Journal of Pain and Symptom Management led contributions. Keyword analysis highlighted research foci on deep learning, neural networks, quality-of-life enhancement, survival prediction, AI model development, and clinical optimization. Emerging trends emphasize machine learning and holistic AI integration.

**Conclusion:**

Despite the increasing number of related studies in recent years, the field remains in its early developmental stage, indicating vast potential for further research. Studies have shown that international collaboration, particularly between the United States and China, is crucial for enhancing global academic influence. Prominent institutions in the United States, such as Harvard Medical School and the University of Pennsylvania, have led research in this area, while the involvement of other countries, especially developing nations, still requires strengthening. Technological analyses reveal that machine learning, deep learning, and natural language processing are becoming increasingly significant in palliative care. Future research will focus on improving patient quality of life, personalized treatment, and disease prognosis prediction, with an emphasis on interdisciplinary collaboration and the integration of technology with clinical practice to foster the innovative development of artificial intelligence in palliative care.

**Systematic review registration:**

https://osf.io/, identifier https://doi.org/10.17605/OSF.IO/YCHNQ.

## 1 Introduction

The World Health Organization (WHO) defines palliative care as an approach that aims to enhance the quality of life for patients and their families who are facing life-threatening illnesses (1). Its primary objective is to provide support for both patients and their families in managing the multifaceted challenges posed by serious diseases, through the alleviation of suffering and the effective management of symptoms. Central to palliative care is the provision of comprehensive support that addresses the physical, psychological, social, and spiritual needs of patients, underscoring the importance of interdisciplinary collaboration. This collaboration involves a diverse range of healthcare professionals, including physicians, nurses, social workers, psychologists, and spiritual advisors, all working together to develop a personalized care plan for each patient (2).

Palliative care was first introduced by Cicely Saunders in the United Kingdom, who founded the renowned St. Christopher’s Hospice in 1967 to offer comfort and care to patients in the terminal stages of life (3). Since its inception, the concept of palliative care has evolved into a globally recognized model of care, marking a shift in the traditional medical paradigm. While earlier models predominantly focused on curative treatments, palliative care emphasizes a patient-centered approach, prioritizing not only the management of physical symptoms but also emotional and spiritual support. This holistic approach reflects a more humane and compassionate model of care. Clinical research has consistently demonstrated that palliative care significantly enhances patients’ quality of life, reduces hospital admissions, lowers healthcare costs, and increases overall satisfaction for both patients and their families (4).

With the rising prevalence of chronic diseases, such as cardiovascular diseases, diabetes, and neurodegenerative disorders, managing long-term treatment has become increasingly complex. These conditions, often chronic and incurable, impose substantial physical, psychological, and economic burdens on patients (5). In this context, palliative care plays a critical role in managing chronic illnesses by providing multidimensional symptom control, psychological support, and optimizing overall quality of life. It has become an essential intervention in alleviating both the physical and psychological burdens on patients, while ensuring their dignity remains intact. Given the chronic and often multifaceted nature of these diseases, and the limitations of curative therapies, palliative care offers integrated symptom management, pain relief, and psychological assistance, thereby enabling patients to maintain a high quality of life throughout their illness trajectory. Furthermore, palliative care helps ease the psychological burden on families and contributes to reducing unnecessary healthcare resource utilization, mitigating overtreatment, and positively influencing health economics by reducing overall healthcare expenditure (6). Additionally, palliative care plays a pivotal role in clinical decision-making, preserving patient autonomy by integrating their preferences into complex medical decisions and ensuring that treatment strategies are consistent with their values and expectations for life.

Despite significant global advancements in palliative care, numerous challenges remain, including resource constraints, a shortage of adequately trained professionals, and the difficulty of providing individualized care that addresses the unique needs of each patient. Palliative care inherently requires close collaboration among interdisciplinary teams; however, many regions, particularly those in low- and middle-income countries, experience a critical lack of trained personnel and limited access to specialized training resources. This results in inconsistent service coverage and significant variability in the quality of care provided. Furthermore, the complex and often fluctuating nature of symptoms experienced by palliative patients poses considerable challenges in the continuous monitoring and real-time analysis of their conditions, hindering the development of tailored care plans. As the volume and complexity of medical data increase, effectively integrating and utilizing this information to optimize treatment strategies and enhance patient outcomes remains a significant hurdle in the ongoing evolution of palliative care (7).

The rapid development of Artificial Intelligence (AI) in the healthcare sector presents new opportunities for transforming medical care models (8). Leveraging technologies such as deep learning and natural language processing, AI is capable of processing and analyzing large volumes of medical data, thereby assisting healthcare providers in making more accurate diagnoses and intervention decisions. Additionally, AI has the potential to improve the equitable distribution of healthcare resources, particularly in underserved and remote areas, enhancing the accessibility and availability of healthcare services (9).

The application of AI in palliative care is emerging as an important and promising research direction, particularly in areas such as enhancing patient comfort, supporting end-of-life decision-making, and optimizing care processes (10). AI is currently employed to assess changes in patient conditions and predict end-of-life risks using advanced data analysis and machine learning algorithms, enabling the development of personalized treatment and care plans. Furthermore, AI technologies support healthcare professionals in offering emotional support, alleviating patient suffering, and optimizing resource allocation and management, thus ensuring the sustainability and efficiency of palliative care services (11). These technologies not only improve the accuracy and timeliness of treatment but also provide intelligent decision support for both families and healthcare providers, ensuring the dignity and quality of life of the patient are preserved (12). As a result, AI applications have the potential to significantly enhance the effectiveness of palliative care, offering a promising technological foundation for future advancements and innovations in this field.

Although research on the application of AI in palliative care has been growing in recent years, the landscape of this research remains unclear, and there is a lack of systematic and comprehensive visual analysis. This gap hinders a full understanding of the progress and potential future directions of AI in palliative care, thereby limiting future research and practical implementation. Therefore, a bibliometric analysis of AI applications in palliative care is essential to bridge this gap.

Bibliometrics, a quantitative approach to analyzing academic literature, provides valuable insights into the evolution of academic research, development trends, and emerging research hotspots by statistically analyzing academic publications (13). This method allows researchers to examine literature distribution, author collaboration networks, and interdisciplinary connections, facilitating the identification of innovative research directions and potential gaps within the field. By constructing data models, bibliometrics can assess the quantity, impact, and dissemination of academic resources, offering researchers a measurable and objective perspective to identify frontier issues and predict future research trends. Ultimately, bibliometrics provides scientific evidence to guide both research and policymaking in relevant areas (14).

This study aims to evaluate the current state and development trends of AI research in palliative care through bibliometric analysis. By identifying key technologies, research hotspots, and future directions, this study will offer clear research pathways and actionable recommendations for future investigations. This will help promote the innovative application of AI in palliative care and further advance both academic research and clinical practice in the field.

The study protocol has been preregistered on the Open Science Framework,^1^ with the doi: https://doi.org/10.17605/OSF.IO/YCHNQ.

## 2 Data and methods

### 2.1 Data sources and search strategy

The relevant literature was systematically retrieved from the Web of Science Core Collection (WoSCC), a comprehensive and highly respected global scientific database (15), covering the period from its inception up to February 28, 2025. The search strategy integrated an extensive array of relevant terms, including synonyms and alternative expressions, thus ensuring an exhaustive exploration of the research topic. The search terms are as follows: (“Artificial Intelligence” OR “AI” OR “Machine Learning” OR “Deep Learning” OR “Neural Network*” OR “Computer Vision” OR “Expert System*” OR “Cognitive Computing” OR “Reinforcement Learning” OR “Supervised Learning” OR “Unsupervised Learning” OR “Data Mining” OR “Big Data” OR “Explainable AI” OR “AI Application*” OR “AI Model*” OR “Generative AI”) AND (“Palliative Care” OR “Hospice Care” OR “Palliative Nursing” OR “Advance Care Planning” OR “Palliative Medicine” OR “palliat*” OR “End of life” OR “EOL” OR “Comfort Care” OR “Terminal Illness” OR “Terminally Ill” OR “Terminal Phase” OR “Stage IV cancer” OR “Life-limiting Illness” OR “Advanced Illness” OR “Supportive Care”).

Inclusion criteria: Original research on the application of AI in the field of palliative care.

Exclusion criteria:

1. Conference papers, dissertations, letters, book chapters, and duplicate publications.
2. Studies involving secondary research, such as reviews, commentaries, or conceptual papers.
3. Publications with incomplete information, including unclear research designs or insufficient details regarding interventions.
4. Articles published in languages other than English, given the available resources within the review team and the time and cost constraints associated with translation.

For duplicate publications, only the earliest and most comprehensive version was included.

**FIGURE 1:**
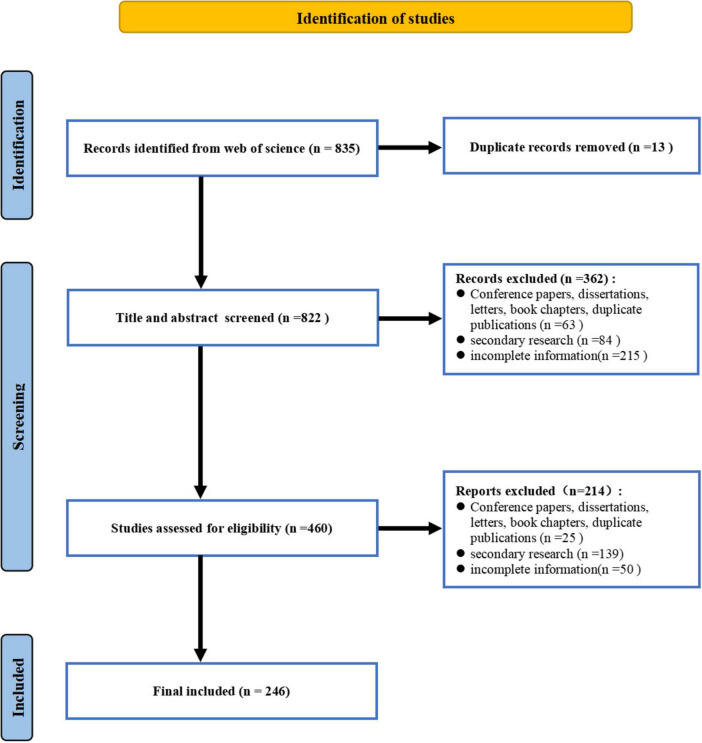
Flowchart of the literature-screening process.

The abstracts of the identified articles were uploaded into the Covidence web application,^2^ where duplicates were systematically removed. Following this, the titles and abstracts of the articles were independently screened by two reviewers (MP and RH). Each article was subsequently classified as either “include,” “exclude,” or “potentially include.” In cases of disagreement, a third reviewer (HL) was consulted to resolve the discrepancies. Studies that successfully passed the initial screening stage advanced to a full-text review, during which two authors (MP and RH) independently evaluated each study. Any remaining discrepancies were addressed through in-depth discussions between the authors. A detailed overview of the study selection process is provided in Figure 1.

### 2.2 Data analysis and network mapping

In this study, an extensive bibliometric analysis was conducted employing three specialized software tools, namely HistCite, VOSviewer, and CiteSpace, each specifically designed to address distinct analytical objectives. Initially, HistCite, a sophisticated citation analysis tool conceived by Eugene Garfield, the pioneer behind the Science Citation Index (SCI), was employed to delineate the historical trajectory of citations within the domain. HistCite facilitates the identification of pivotal authors, journals, and seminal publications, thereby offering a comprehensive perspective on the field’s developmental chronology (16). For this study, HistCite Pro 2.1 was utilized to generate structured tables that meticulously charted the literature’s progression, elucidating the field’s evolutionary dynamics over time. Subsequently, VOSviewer, a Java-based application developed by the Center for Science and Technology Studies at Leiden University, was deployed for the mapping and visualization of scientific knowledge. Renowned for its proficiency in managing extensive bibliometric datasets, VOSviewer excels in generating diverse network analyses, including co-occurrence networks, citation networks, and term frequency analyses (17). In this research, VOSviewer 1.6.20 was employed to scrutinize collaboration networks and visualize keyword co-occurrence, thereby uncovering central themes, prevailing trends, and critical publications within the field. Finally, CiteSpace, an open-source software developed by Chaomei Chen, was utilized to conduct an in-depth analysis of the scientific literature. This tool is particularly adept at constructing science maps that reveal research hotspots, foundational knowledge, and emergent trends (18). In this study, CiteSpace 6.3.R1 was employed to detect keyword bursts, thereby identifying and delineating the evolving research trends within the field. Collectively, these tools provided a robust framework for a comprehensive bibliometric evaluation, offering nuanced insights into the field’s historical development, collaborative networks, and emerging research foci.

### 2.3 Ethical considerations

The data utilized in this study were sourced from the WoSCC, and no involvement from patients or public contributors was included in this research.

## 3 Results

### 3.1 Analysis of publication outputs and the total local citation score

**FIGURE 2:**
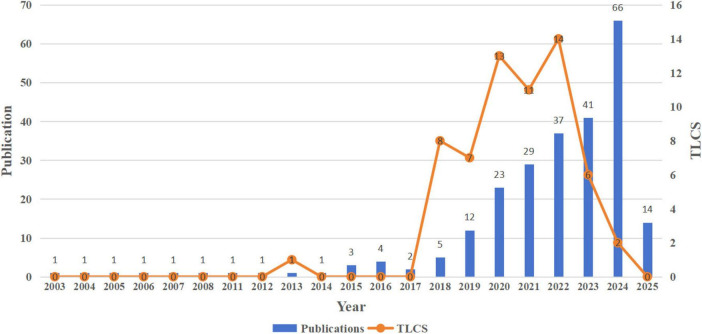
Publication output and the TLCS over time. TLCS, total local citation score.

A total of 835 articles were retrieved from the WoSCC. Following a comprehensive data cleaning process, 246 articles (29.46%) were retained for further analysis. The trends in the annual publication volume (depicted by blue bars) and citation counts (Total Local Citation Score, TLCS, represented by the orange line) of the applications of AI in palliative care are illustrated in Figure 2. Table 1 presents the annual publication volume, TLCS, and Total Global Citation Score (TGCS) for the included studies. From 2003 to 2014, the annual publication volume remained stable, albeit at a relatively low level, with only one publication per year. Beginning in 2015, the number of publications began to rise. This upward trend continued after 2020, culminating in a peak of 66 publications in 2024. As the search period was limited to February 28, 2025, the publication count for 2025 stands at 14. The TLCS, a bibliometric metric that measures the number of citations a paper receives within a specific research domain or local dataset, is calculated by the total number of direct citations a paper receives from other papers within the defined scope. Conversely, TGCS refers to the cumulative total of citations a research paper receives globally, reflecting its academic influence and dissemination (19).

**TABLE 1:** Annual publication volume, TLCS, and TGCS.

Rank	Publication year	Publications	TLCS^a^	TGCS^b^
1	2003	1	0	23
2	2004	1	0	0
3	2005	1	0	2
4	2006	1	0	15
5	2007	1	0	134
6	2008	1	0	3
7	2011	1	0	33
8	2012	1	0	20
9	2013	1	1	32
10	2014	1	0	14
11	2015	3	0	4
12	2016	4	0	117
13	2017	2	0	109
14	2018	5	8	300
15	2019	12	7	553
16	2020	23	13	810
17	2021	29	11	654
18	2022	37	14	392
19	2023	41	6	223
20	2024	66	2	116
21	2025	14	0	1

^a^TLCS, total local citation score.

^b^TGCS, total global citation score.

In terms of TLCS, from 2003 to 2017, citation counts were almost negligible, with only a single citation recorded in 2013. However, in 2018, the citation count rose to 8, followed by 7 citations in 2019, 13 in 2020, 11 in 2021, and 14 in 2022. Notably, the citation count declined to 6 in 2023, and further dropped to 2 in 2024. Regarding TGCS, citation counts remained relatively low prior to 2015, with a solitary citation of 134 recorded in 2007. However, after 2016, citations began to rise significantly, and the TGCS consistently exceeded 100 from 2016 to 2024, reaching a peak of 810 in 2020.

### 3.2 Analysis of countries/regions

**FIGURE 3:**
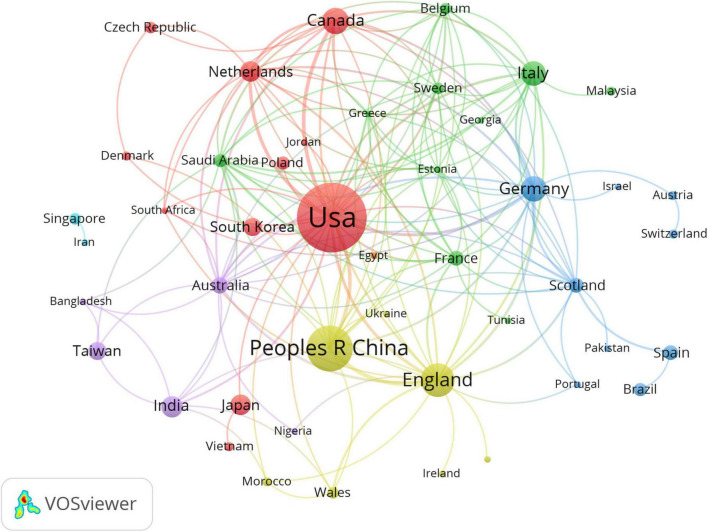
Visualization of research networks of countries/regions.

A total of 45 countries/regions contributed to the publication of 246 articles. Figure 3 illustrates the national co-occurrence network of artificial intelligence applications in palliative care. Each node represents a country, with the size of the node proportional to the frequency of its appearance in the relevant literature. Larger nodes indicate higher publication volumes in the field (20). In this figure, the United States and China emerge as the most prominent nodes, followed by countries such as Canada, Germany, and England, highlighting their significant presence in the literature on this topic. The connecting lines between nodes represent collaborative relationships between countries, with the line thickness corresponding to the strength of the collaboration (21). For instance, the thicker lines between the United States and countries like Canada, Netherlands, China, and England indicate strong collaborations, while thinner lines between the United States and countries like Japan, Morocco, and Wales suggest less frequent cooperation. Additionally, the line between China and England is relatively thick, indicating a closer collaboration between these two nations in this field. Table 2 lists the top 10 countries in terms of their application of artificial intelligence in palliative care, showing their publication counts, TLCS, and TGCS. According to the table, the United States ranks first with 105 publications and leads in both TLCS (51) and TGCS (1988). China ranks second with 45 publications; although its publication count is lower than that of the United States, its TGCS (521) is relatively high. England holds the third position with 29 publications and a TGCS of 453, which, despite a smaller number of publications, indicates significant global influence. Canada ranks fourth, with only 15 publications but a relatively high TLCS (3). Germany ranks fifth, with 14 publications, a TLCS of 0, and a TGCS of 79. Italy comes sixth, with 13 publications and a high TGCS of 132. India ranks seventh with 10 publications, and despite the lower quantity, its TGCS (40) remains relatively high. The Netherlands ranks eighth with 9 publications and a TGCS of 47. Japan ranks ninth with 9 publications and a lower TGCS (39). Finally, Taiwan occupies the tenth position, with 7 publications and a relatively high TGCS of 50.

**TABLE 2:** Top 10 countries/regions by number of publications.

Rank	Country	Publications	TLCS^a^	TGCS^b^
1	USA	105	51	1,988
2	China	45	8	521
3	UK	29	0	453
4	Canada	15	3	133
5	Germany	14	0	79
6	Italy	13	1	132
7	India	10	0	40
8	Netherlands	9	0	47
9	Japan	9	4	39
10	Taiwan	7	0	50

^a^TLCS, total local citation score.

^b^TGCS, total global citation score.

### 3.3 Analysis of institutions

**FIGURE 4:**
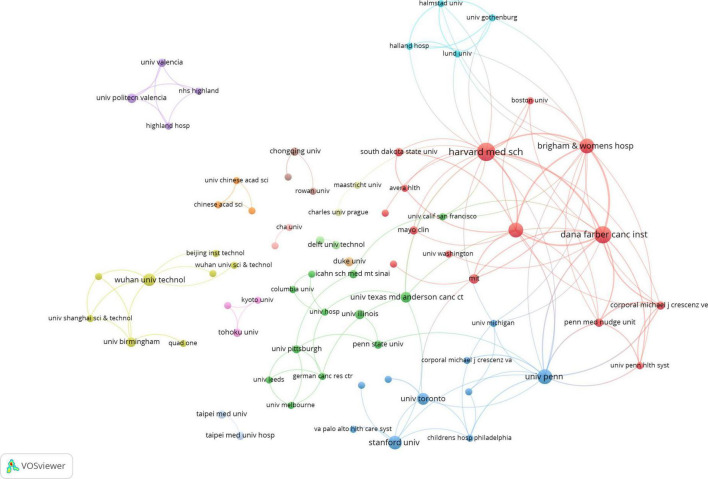
Visualization of research networks of institutions.

A total of 615 institutions contributed to the publication of 246 papers. Figure 4 illustrates a co-occurrence map of institutional collaborations, where each node corresponds to an institution. The size of each node is proportional to the frequency of the institution’s occurrence in the literature (22). Notably, prominent institutions such as Harvard Medical School, Dana-Farber Cancer Institute, and the University of Pennsylvania are represented by larger nodes, reflecting their substantial Contribution To The Field. The connecting lines between the nodes represent the collaborative relationships among institutions, with the line thickness indicating the frequency of these collaborations (23). For example, the thicker connecting lines between Harvard Medical School, Dana-Farber Cancer Institute, and Brigham and Women’s Hospital signify a high level of collaborative activity among these institutions. Table 3 presents the top 10 institutions by publication volume, with eight of these institutions located in the United States. Harvard Medical School leads the rankings with 16 publications, boasting a TLCS of 5 and a TGCS of 311. Following closely is the Dana-Farber Cancer Institute, which published 14 papers and holds the highest TGCS of 424. Other notable institutions, including the University of Pennsylvania, Brigham and Women’s Hospital, and Massachusetts General Hospital, each contributed 10 papers. Despite publishing only 8 papers, Stanford University ranks highly due to its remarkable TGCS of 410. Furthermore, Wuhan University of Technology published 7 papers, with a TGCS of 39. Other institutions, such as the University of Toronto, University of Texas MD Anderson Cancer Center, and Massachusetts Institute of Technology, published 6, 5, and 4 papers, respectively.

**TABLE 3:** Top 10 institutions by number of publications.

Rank	Institution	Publications	TLCS^a^	TGCS^b^
1	Harvard Medical School (USA)	16	5	311
2	Dana-Farber Cancer Institute (USA)	14	14	424
3	University of Pennsylvania (USA)	10	15	371
4	Brigham and Women’s Hospital (USA)	10	12	268
5	Massachusetts General Hospital (USA)	10	10	203
6	Stanford University (USA)	8	7	410
7	Wuhan University of Technology (China)	7	0	39
8	University of Toronto (Canada)	6	1	55
9	University of Texas MD Anderson Cancer Center (USA)	5	2	47
10	Massachusetts Institute of Technology (USA)	4	6	191

^a^TLCS, total local citation score.

^b^TGCS, total global citation score.

### 3.4 Analysis of journals

A total of 151 journals contributed to the publication of 246 papers. As illustrated in Table 4, the *Journal of Pain and Symptom Management* leads with 20 publications, reporting a TLCS of 8 and TGCS of 156. Following closely, the *Journal of Palliative Medicine* ranks second, with 12 articles published, a TLCS of 8, and a TGCS of 166. The *Scientific Reports* holds the third position, publishing 8 articles, although it records a TLCS of 0 and a TGCS of 19. The *Journal of Cleaner Production* and *Sustainability* each contributed 5 articles. The former achieved a TLCS of 2 and a TGCS of 171, while the latter, despite having the same publication count, reported a TLCS of 0 and a TGCS of 31. In sixth place, *BMC Palliative C*are also published 5 articles, but with a TLCS of 0 and a TGCS of 20. The *Journal of the American Medical Informatics Association* published 4 articles, attaining a TLCS of 4 and a TGCS of 40. Similarly, the *Journal of Energy Storage* published 4 articles, yet its TLCS stands at 0, with a TGCS of 38. The *American Journal of Hospice & Palliative Medicine* contributed 4 articles as well, registering a TLCS of 0 and a TGCS of 28. Finally, *JAMA Network Open* secured the tenth position, with 3 articles published, a TLCS of 0, and a remarkable TGCS of 216.

**TABLE 4:** Top 10 journals that published papers on the application of AI^a^ in palliative care.

Rank	Journal	Publications	TLCS^a^	TGCS^b^
1	Journal of Pain and Symptom Management	20	8	156
2	Journal of Palliative Medicine	12	8	166
3	Scientific Reports	8	0	19
4	Journal of Cleaner Production	5	2	171
5	Sustainability	5	0	31
6	BMC Palliative Care	5	0	20
7	Journal of the American Medical Informatics Association	4	4	40
8	Journal of Energy Storage	4	0	38
9	American Journal of Hospice and Palliative Medicine	4	0	28
10	JAMA Network Open	3	0	216

^a^TLCS, total local citation score.

^b^TGCS, total global citation score.

### 3.5 Analysis of authors and coauthorship networks

**FIGURE 5:**
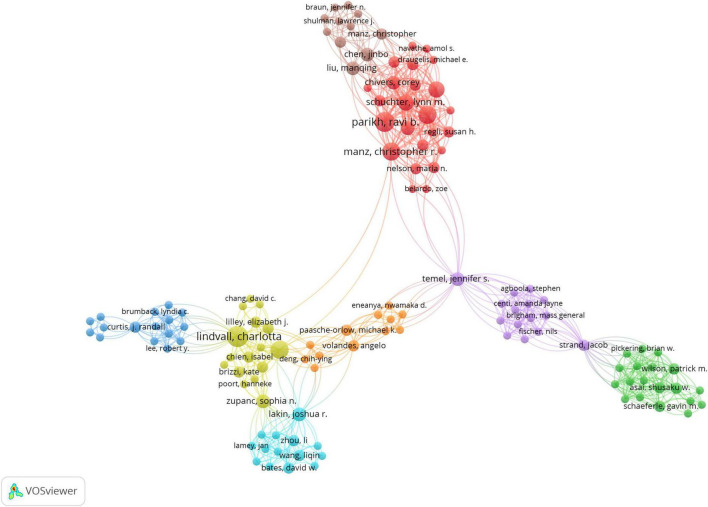
Visualization of research networks of authors.

A total of 1,456 authors contributed to the publication of 246 articles. Table 5 presents the top 10 authors ranked by their publication volume, along with their respective TLCS and TGCS. In terms of publication output, Parikh RB and Lindvall C are tied for the highest position, each having authored 7 publications, underscoring their substantial productivity in this domain. Closely following them is Patel MS, with 6 publications, while authors ranked 4th through 10th have published 5 or fewer papers, suggesting a relatively lower level of research output. Concerning TLCS, Parikh RB, Patel MS, and Manz CR each achieved a top score of 15, highlighting their significant academic impact within the field. This score reflects not only the quality of their research but also the widespread recognition by their peers. When considering TGCS, Parikh RB leads with 361 points, further corroborating the global prominence and citation frequency of his work. Patel MS closely follows with 360 points, indicating that his research also commands substantial international recognition. Figure 5 illustrates the author collaboration network, offering a visual representation of the interconnectedness between authors and their academic influence. The size of each node in the network typically correlates with an author’s scholarly activity and impact; larger nodes represent higher research output or greater academic influence within the field (24). For example, the prominent nodes of Parikh RB and Manz CR reflect their significant academic presence. The edges, or connections, between nodes signify collaborative relationships, with the thickness of these lines denoting the frequency and intensity of these collaborations (25). Notably, the thick connection between Parikh RB and Manz CR indicates a frequent and close partnership. Additionally, Schuchter LM and Shulman LN demonstrate a clear collaborative link. The figure also highlights distinct clustering patterns, where groups of authors form relatively independent research clusters. These clusters typically consist of researchers working within similar or related fields, collaborating on shared research topics. This organization may reflect the major research trends and directions within the discipline (26). For example, Lindvall C’s research group clusters with Chang David C, Lilley Elizabeth J, and others, suggesting a focused collaboration on overlapping research themes. Finally, the central nodes in the collaboration network often represent key scholars within the field. These scholars not only exert significant influence within the discipline but also serve as vital connectors between disparate research groups (27). In this context, Parikh RB and Manz CR are prime examples of central nodes, as they link multiple distinct research clusters, further solidifying their pivotal roles in the academic landscape.

**TABLE 5:** Top 10 authors who published research papers.

Rank	Author	Publications	TLCS^a^	TGCS^b^
1	Parikh RB	7	15	361
2	Lindvall C	7	9	205
3	Patel MS	6	15	360
4	Schuchter LM	5	14	352
5	Shulman LN	5	14	352
6	Manz CR	5	15	205
7	Tulsky JA	5	8	166
8	Regli SH	4	14	346
9	Chivers C	4	10	333
10	Shah NH	4	6	206

^a^TLCS, total local citation score.

^b^TGCS, total global citation score.

### 3.6 Co-occurrence of keywords

**FIGURE 6:**
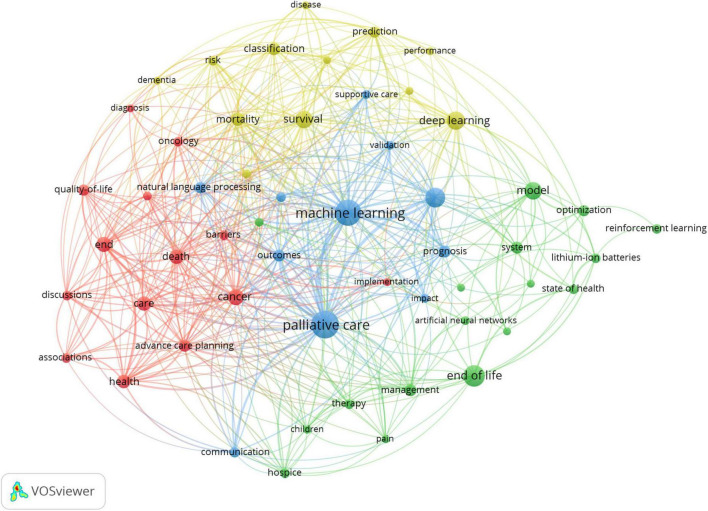
Keyword co-occurrence network.

Figure 6 presents the co-occurrence network of keywords pertinent to the subject under investigation. The central node, “machine learning,” which is characterized by its larger size and robust connections with surrounding nodes, underscores its central and pivotal role within the realm of palliative care. In particular, closely associated nodes, including “deep learning,” “model,” and “artificial neural networks,” reflect the growing prominence of advanced AI technologies, particularly in the domains of data analysis and predictive modeling in palliative care. The nodes “palliative care” and “end of life,” which are comparatively larger and tightly interconnected, emphasize the critical importance of palliative care in the context of patients nearing the end of life. These nodes demonstrate strong linkages with other key terms such as “care,” “cancer,” and “quality of life.” The varied colors of the nodes represent distinct thematic clusters within the network (28). Specifically, the red nodes, which encompass terms such as “death,” “discussions,” and “advance care planning,” are indicative of the sensitive and nuanced decision-making processes, as well as the need for effective communication, in the provision of end-of-life care. On the other hand, the green nodes, including “optimization,” “reinforcement learning,” and “system,” highlight the application of AI in optimizing systems and enhancing overall performance within the field. Meanwhile, the blue nodes, such as “natural language processing” and “prognosis,” underscore the significant role of AI in the analysis and interpretation of patient data, particularly through the use of natural language processing (NLP) techniques and the prediction of patient prognosis. Furthermore, the thickness of the connecting lines between nodes serves as a visual representation of the strength of their co-occurrence. Notably, the lines connecting “machine learning” to the nodes “palliative care,” “model,” and “deep learning” are relatively thick, reflecting strong associations among these concepts. Likewise, the connection between “palliative care” and “communication” is also characterized by a thicker line, further indicating the substantial interrelationship between these key elements within the broader context of palliative care.

### 3.7 Burst keyword detection

**FIGURE 7:**
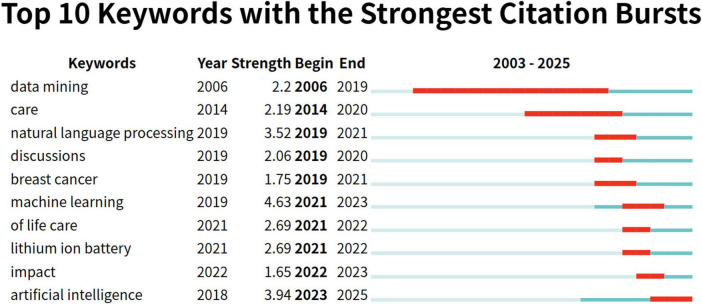
Top 15 keywords with the strongest citation bursts.

Keyword emergence is a method employed to monitor and quantify the temporal or condition-specific fluctuations in the prevalence of particular keywords (29). This technique is essential for detecting emerging trends, identifying prominent research topics, or pinpointing potential avenues for future investigation (30). Through the analysis of keyword emergence mapping, researchers can delineate active research areas and evolving trends within a specific discipline. This is exemplified by a marked increase in the frequency of certain keywords, represented by the red bars, over different years. As demonstrated in Figure 7, the top 10 emergent keywords in the field are listed, with “machine learning” exhibiting the highest burst intensity (4.63), followed by “artificial intelligence” (3.94) and “natural language processing” (3.52). Additionally, “data mining” (13 years) stands out as the keyword with the longest duration of burst activity. Notably, “artificial intelligence” has emerged as the most dominant keyword in recent years, as reflected in the keyword mapping.

## 4 Discussion

### 4.1 Principal findings

Based on the analysis of annual publication trends, AI research in palliative care has significantly developed, with a steady increase in publications since 2018, peaking at 66 articles by 2024. However, annual output has not yet surpassed 100 articles, indicating that the field remains in an exploratory phase, with considerable room for growth in academic frameworks and knowledge accumulation. To enhance research impact, optimization strategies are necessary. First, focusing on key clinical issues and integrating advanced AI technologies such as deep learning can improve clinical relevance (31). Second, fostering interdisciplinary collaboration across clinical medicine, information science, and nursing will elevate scientific rigor. Third, leveraging professional journals and creating academic communities will strengthen dissemination efforts. Although TLCS values are limited, TGCS values have risen, reflecting a growing global impact, highlighting the need for international collaborations to further expand research influence (32).

The analysis of publication volume, TLCS, and TGCS reveals that the United States leads globally in AI and palliative care research, with the highest values across these metrics, indicating its dominance in both research scale and impact. This suggests that nations with established research systems and robust international collaborations are more likely to drive breakthroughs in emerging interdisciplinary fields, significantly influencing global academic progress (33). Both the United States and China are key roles in international collaboration, contributing substantially to the field and reinforcing their leadership in AI and palliative care research. The United Kingdom, Canada, and Germany also hold influential positions within the global network, underlining their importance. Europe, particularly Germany and Italy, exhibits a strong regional collaboration framework, while the partnership between the United States and South Korea stands out for its intensity and sustained efforts. In contrast, intra-Asian collaboration, as seen between Japan and Vietnam, remains limited, suggesting untapped potential for cooperation. Cluster analysis reveals distinct regional patterns: North American-European ties (red cluster), Europes limited, suggesting untappreen cluster), a more independent approach in France and South Korea (blue cluster), and the emerging Asian network between China and India (yellow cluster). This study highlights the need for increased collaboration between the United States, China, and other countries to foster global innovation, with Europed cluster), Europes limited, suggesting untappreen cluster), a mouraged to enhance intra-regional and international partnerships. The regional nature of the collaboration network emphasizes the need for cross-regional cooperation to advance sustainable AI applications in palliative care (34).

Research on the application of AI in palliative care has predominantly been led by institutions in the United States, with eight out of the top 10 contributors located there, underscoring the country’s dominant role. This highlights a disparity, as developing nations, especially in the global South, remain underrepresented. The research landscape exhibits a core-periphery structure, with leading institutions such as Harvard Medical School, Dana-Farber Cancer Institute, and the University of Pennsylvania forming tightly interconnected hubs of collaboration. Notably, Harvard and Dana-Farber’s close cooperation emphasizes their centrality. In contrast, institutions like Wuhan University of Technology in China, though contributing to the field, show limited international impact due to fewer publications and lower citation scores. This peripheral positioning suggests limited collaboration with core institutions. To foster global equity, enhancing cooperation between core and peripheral institutions, particularly in data sharing and joint research, is crucial. Furthermore, increasing involvement from institutions in developing countries can strengthen their global research influence.

Besides, the study, through a visual analysis of the author collaboration network, identifies key structural patterns in artificial intelligence research in palliative care. Scholars Manz, Christopher, and Parikh, Ravi B emerge as central figures within the network, with significantly larger nodes and extensive connections to other researchers, indicating their leadership and academic influence in the field. The network reveals distinct interdisciplinary and interinstitutional characteristics, highlighting an integration trend across diverse scientific areas. Such collaboration fosters knowledge exchange, innovation, and the development of multidimensional research perspectives, which are crucial for solving complex medical challenges (35). The findings suggest that a singular disciplinary approach is inadequate for addressing the growing complexity of clinical problems, emphasizing the importance of multidisciplinary synergy and interinstitutional collaboration as key drivers of innovation in this field (36). This approach accelerates the clinical translation of research and opens new avenues for future research, strengthening academic collaboration and communication.

In the realm of palliative care, AI, particularly machine learning, has emerged as a pivotal research focus, driving advancements in several key areas. The integration of deep learning, artificial neural networks, and predictive models showcases the potential of AI technologies in disease prediction, diagnosis, and personalized treatment planning. Clinical studies have demonstrated that deep learning and neural networks enhance the accuracy of disease prediction, facilitating the development of tailored treatment regimens that significantly improve patient outcomes and quality of life (37). Research in palliative care emphasizes leveraging AI to enhance end-of-life care, focusing on improving patient comfort and care management through intelligent methodologies. AI technologies have been shown to alleviate suffering, optimize care plans, and ultimately improve the quality of life for terminal patients (38). Machine learning also plays a crucial role in survival prognosis prediction, enabling more accurate survival assessments through large dataset analysis, which informs personalized care plans and supports more informed clinical decisions (39). Additionally, studies highlight the importance of constructing and validating predictive models to ensure their clinical applicability. The strong relationship between “risk” and “health” further underscores AI’s value in identifying potential health risks, particularly in complex diseases like cancer, thereby optimizing treatment pathways and improving clinical outcomes (40). NLP also plays a growing role in enhancing communication and decision-making in palliative care. By analyzing patient-provider interactions, NLP technologies enable healthcare providers to better understand patient needs, delivering more targeted interventions and emotional support (41). The continuous evolution of AI, including optimization and reinforcement learning, promises further improvements in resource allocation and treatment protocols, boosting efficiency in palliative care. Overall, AI providers dalidating predictive modelisease prediction, patient management, model optimization, and risk assessment, heralding significant advances in patient care and treatment strategies.

The keyword burst analysis reveals a notable evolution in AI research within palliative care, offering valuable insights into emerging trends and future directions. Early research, focused on “data mining,” showed significant burst intensity between 2006 and 2019, emphasizing the extraction of valuable information from vast healthcare datasets. This foundational work enabled the subsequent application of AI technologies in the field. Studies highlight that data mining aids in identifying potential health issues from patient records, thereby optimizing personalized treatment plans in palliative care (42). In recent years, the surge in NLP and machine learning research, particularly in 2019 and 2021, signals their growing prominence in palliative care. The rise of NLP is particularly notable, as its applications in patient communication, medical record analysis, and emotional support offer innovative tools for improving care. Clinical studies demonstrate that NLP can extract critical information from medical texts, supporting real-time clinical decision-making and enhancing care quality (43). The keyword “breast cancer” indicates a shift toward AI’s application in specific diseases, particularly for symptom management, prognosis prediction, and personalized care plans. AI has proven valuable in predicting outcomes and guiding treatment for breast cancer patients, improving their quality of life (40). The prominence of “artificial intelligence” suggests continued research growth through 2025, signifying AI’s increasing integration into palliative care. Additionally, the emergence of “impact” as a keyword highlights the importance of evaluating AI technologies’ clinical effects. Research has shown that AI-assisted care plans enhance symptom relief and improve both patient comfort and healthcare efficiency (44). While seemingly unrelated, “lithium ion battery” hints at AIbatterye both patient comfort and healthcd remote monitoring, which could offer crucial support for home-based palliative care (31). Studies confirm that mobile health devices enable real-time patient monitoring, offering flexible palliative care (45). Future research will focus on refining AI technologies and assessing their clinical outcomes, with an expanding focus on personalized care, specific diseases, and quality-of-life improvements (43).

The visual analytics results reveal the current state and trends in AI application within palliative care, highlighting that research in this area is still in its early stages. Despite the widespread integration of AI across diverse medical disciplines, its implementation in palliative care is still limited, facing multiple barriers including ethical and clinical challenges. First, ethical concerns represent one of the foremost obstacles to AI adoption in palliative care (46). Palliative care primarily focuses on alleviating patient suffering and enhancing quality of life. In this context, it is crucial to maintain patient autonomy and privacy when addressing sensitive issues. Therefore, ensuring that AI technologies align with the humanistic values fundamental to palliative care remains a central concern. Striking a balance between the efficiency of AI and the compassion inherent in human-centered care is an area requiring focused attention in future research (47). Moreover, challenges at the clinical practice level further hinder the integration of AI into palliative care. The successful application of AI demands interdisciplinary collaboration among experts in medicine, engineering, and ethics. However, the current lack of adequate integration and coordination across these fields limits the potential of AI in this domain. Additionally, Palliative care teams, which are primarily made up of professionals with significant experience in emotional support and clinical decision-making, may be hesitant to adopt AI technologies due to concerns about their adaptability and utility (48). Compounding these issues are challenges such as incomplete data, technological instability, and high costs, which further restrict the widespread adoption of AI. Thus, future research should prioritize overcoming these ethical and clinical barriers. On one hand, it is essential to explore how to reconcile AI with the human-centered approach that defines palliative care. On the other hand, strengthening interdisciplinary collaboration to better integrate AI technologies into existing palliative care models will enhance their operational feasibility and clinical applicability.

### 4.2 Limitations

The results may be subject to some inherent bias since only the WoSCC database was searched. However, it is worth highlighting that WoS is one of the most influential multidisciplinary academic literature indexing platforms globally, renowned for its authority and broad coverage. WoS includes over 12,400 high-impact academic journals from various parts of the world, covering core literature across numerous disciplines (49). The inclusion of these high-quality journals and papers ensures reliable and comprehensive data support for this study. Furthermore, WoSCC offers a powerful set of citation analysis tools that enable in-depth exploration of academic impact and development trends within specific research fields. These tools provide valuable insights into current research hotspots, assisting scholars in understanding the evolution of their fields and offering useful references for future research endeavors.

## 5 Conclusion

This study provides a visualized analysis of the application of AI in the field of palliative care, revealing the growth trends and future developmental potential of research in this domain. Despite the increasing volume of publications year by year, the research remains in its early stages, indicating a vast space for further exploration. The findings demonstrate that international collaboration has played a pivotal role in advancing global academic impact, particularly highlighting the increasingly close cooperation between the United States and China. In terms of institutional collaboration, major institutions in the United States, such as Harvard Medical School and the University of Pennsylvania, have been leading the research in this domain. However, the participation of other countries, especially those in the developing world, still requires strengthening. Keyword analysis highlights the central role of technologies such as machine learning, deep learning, and natural language processing in palliative care. Future research will likely focus on enhancing patient quality of life, personalized treatment plans, and disease prognosis prediction. Furthermore, the study suggests that future research directions should emphasize interdisciplinary collaboration, fostering the refined application of technology and its deeper integration with clinical practice. In particular, the prospects for applications in specific disease areas and mobile healthcare should be explored to further promote the deep development and innovative applications of AI in palliative care. Besides, future research should focus on overcoming ethical and clinical practice barriers by balancing AI with the human-centered approach of palliative care and strengthening the integration of AI technologies with existing models through interdisciplinary collaboration.

## Data Availability

The original contributions presented in this study are included in this article/supplementary material, further inquiries can be directed to the corresponding authors.

## Footnotes

1
https://osf.io/

2
https://www.covidence.org
